# Stereotactic ablative body radiotherapy (SABR) combined with immunotherapy (L19-IL2) versus standard of care in stage IV NSCLC patients, ImmunoSABR: a multicentre, randomised controlled open-label phase II trial

**DOI:** 10.1186/s12885-020-07055-1

**Published:** 2020-06-15

**Authors:** Relinde I. Y. Lieverse, Evert J. Van Limbergen, Cary J. G. Oberije, Esther G. C. Troost, Sine R. Hadrup, Anne-Marie C. Dingemans, Lizza E. L. Hendriks, Franziska Eckert, Crispin Hiley, Christophe Dooms, Yolande Lievens, Monique C. de Jong, Johan Bussink, Xavier Geets, Vincenzo Valentini, Giuliano Elia, Dario Neri, Charlotte Billiet, Amir Abdollahi, David Pasquier, Pierre Boisselier, Ala Yaromina, Dirk De Ruysscher, Ludwig J. Dubois, Philippe Lambin

**Affiliations:** 1 The D-Lab and The M-Lab, Department of Precision Medicine, GROW - School for Oncology and Developmental Biology, Maastricht University, Maastricht, The Netherlands; 2 Department of Radiation Oncology (MAASTRO), GROW - School for Oncology and Developmental Biology, Maastricht University Medical Center, Maastricht, The Netherlands; 3 Department of Radiotherapy and Radiation Oncology, Faculty of Medicine and University Hospital Carl Gustav Carus at Technische Universität Dresden, Fetscherstrasse 74, 01307 Dresden, Germany; 4 OncoRay, National Center for Radiation Research in Oncology, Dresden, Germany; 5 Department of Health Technology, Technical University of Denmark, Kongens Lyngby, Denmark; 6 Department of Pulmonary Medicine, Erasmus MC Rotterdam, Rotterdam, The Netherlands; 7 Department of Pulmonary Diseases, GROW - School for Oncology and Developmental Biology, Maastricht University Medical Centre, Maastricht, The Netherlands; 8 Department of Radiation Oncology, University Hospital and Medical Faculty Tübingen, Eberhard Karls University Tübingen, Hoppe-Seyler-Str. 3, 72076 Tübingen, Germany; 9 Cancer Research UK Lung Cancer Centre of Excellence, University College London Cancer Institute, Paul O’Gorman Building, 72 Huntley Street, London, WC1E 6DD UK; 10 Department of Respiratory Diseases, Respiratory Oncology Unit, University Hospitals KU Leuven, Leuven, Belgium; 11 Department of Radiation Oncology, Ghent University Hospital and Ghent University, Ghent, Belgium; 12 Department of Radiation Oncology, Netherlands Cancer Institute—Antoni van Leeuwenhoek Hospital, Plesmanlaan 121, 1066 Amsterdam, CX The Netherlands; 13 Department of Radiation Oncology, Radboud University Medical Center, Nijmegen, The Netherlands; 14 Department of Radiation Oncology, Cliniques Universitaires Saint-Luc, MIRO - IREC Lab, UCL, Bruxelles, Belgium; 15 Dipartimento Diagnostica per Immagini, Radioterapia Oncologica ed Ematologia, Fondazione Policlinico Universitario A. Gemelli IRCCS, Roma, Italy; 16 Università Cattolica del Sacro Cuore, Istituto di Radiologia, Roma, Italy; 17 Philochem AG, Libernstrasse 3, CH-8112 Otelfingen, Switzerland; 18 Department of Chemistry and Applied Biosciences, Institute of Pharmaceutical Sciences, ETH Zurich, Zurich, Switzerland; 19 Department of Radiation Oncology, Iridium Network, Wilrijk (Antwerp), Belgium; 20 University of Antwerp, Faculty of Medicine and Health Sciences, Campus Drie Eiken, Building S, Universiteitsplein 1, 2610 Wilrijk-Antwerp, Belgium; 21 Division of Molecular and Translational Radiation Oncology, Department of Radiation Oncology, Heidelberg Faculty of Medicine (MFHD) and Heidelberg University Hospital (UKHD), Heidelberg Ion-Beam Therapy Center (HIT), 69120 Heidelberg, Germany; 22 Clinical Cooperation Unit Translational Radiation Oncology, National Center for Tumor Diseases (NCT), Heidelberg University Hospital (UKHD) and German Cancer Research Center (DKFZ), Heidelberg, Germany; 23 German Cancer Consortium (DKTK) Core Center, Heidelberg, Germany; 24 Heidelberg Institute of Radiation Oncology (HIRO), National Center for Radiation Oncology (NCRO), Heidelberg University and German Cancer Research Center (DKFZ), Heidelberg, Germany; 25 Academic Department of Radiation Oncology, Oscar Lambret Comprehensive Cancer Center, Lille, France; 26 Department of Radiation Oncology, ICM-Val d’Aurelle, Université de Montpellier, Montpellier, France

**Keywords:** Immunotherapy, L19-IL2, Anti-PD-L1, Anti-PD-1, Radiotherapy, SABR, Phase 2, NSCLC, Stage IV, Multicentre

## Abstract

**Background:**

About 50% of non-small cell lung cancer (NSCLC) patients have metastatic disease at initial diagnosis, which limits their treatment options and, consequently, the 5-year survival rate (15%). Immune checkpoint inhibitors (ICI), either alone or in combination with chemotherapy, have become standard of care (SOC) for most good performance status patients. However, most patients will not obtain long-term benefit and new treatment strategies are therefore needed. We previously demonstrated clinical safety of the tumour-selective immunocytokine L19-IL2, consisting of the anti-ED-B scFv L19 antibody coupled to IL2, combined with stereotactic ablative radiotherapy (SABR).

**Methods:**

This investigator-initiated, multicentric, randomised controlled open-label phase II clinical trial will test the hypothesis that the combination of SABR and L19-IL2 increases progression free survival (PFS) in patients with limited metastatic NSCLC. One hundred twenty-six patients will be stratified according to their metastatic load (oligo-metastatic: ≤5 or poly-metastatic: 6 to 10) and randomised to the experimental-arm (E-arm) or the control-arm (C-arm). The C-arm will receive SOC, according to the local protocol. E-arm oligo-metastatic patients will receive SABR to all lesions followed by L19-IL2 therapy; radiotherapy for poly-metastatic patients consists of irradiation of one (symptomatic) to a maximum of 5 lesions (including ICI in both arms if this is the SOC). The accrual period will be 2.5-years, starting after the first centre is initiated and active. Primary endpoint is PFS at 1.5-years based on blinded radiological review, and secondary endpoints are overall survival, toxicity, quality of life and abscopal response. Associative biomarker studies, immune monitoring, CT-based radiomics, stool collection, iRECIST and tumour growth rate will be performed.

**Discussion:**

The combination of SABR with or without ICI and the immunocytokine L19-IL2 will be tested as 1st, 2nd or 3rd line treatment in stage IV NSCLC patients in 14 centres located in 6 countries. This bimodal and trimodal treatment approach is based on the direct cytotoxic effect of radiotherapy, the tumour selective immunocytokine L19-IL2, the abscopal effect observed distant from the irradiated metastatic site(s) and the memory effect. The first results are expected end 2023.

**Trial registration:**

ImmunoSABR Protocol Code: NL67629.068.18; EudraCT: 2018–002583-11; Clinicaltrials.gov: NCT03705403; ISRCTN ID: ISRCTN49817477; Date of registration: 03-April-2019.

## Background

Lung cancer is the leading cause of cancer-related death worldwide [1, 2], with an estimated mortality of 3.1 million in 2040 [3]. Non-small cell lung cancer (NSCLC) is the most common lung cancer type (85% of cases) and half of these patients have metastatic disease at initial diagnosis [4]. Immune checkpoint inhibitors (ICI), either alone for selected patients (Programmed Cell Death-ligand 1 (PD-L1) ≥ 50% EU and PD-L1 ≥ 1% in USA), or in combination with chemotherapy, have become the standard of care (SOC) for most good performance status (PS) patients with metastatic disease [5]. Metastasized NSCLC patients with oligo-metastatic disease showed a benefit in progression free survival (PFS) when local ablative therapy was added to systemic therapy (chemotherapy ([6–8]) or tyrosine kinase inhibitor ([7, 8])); one trial also demonstrated an improved overall survival (OS) [7]. Oligometastatic disease is usually defined as “limited metastasis” (NCCN guideline [9]), up to three metastases (ESMO guideline [5]) or up to five metastases (European Organization for the Research and Treatment of Cancer (EORTC) lung cancer group consensus definition [10–12] and most clinical trials [13–15]). These guidelines advise to treat these patients with a combination of systemic therapy and local ablative therapy, preferably within a clinical trial.

However, most patients with oligo-metastatic disease will not obtain long-term benefit due to resistance mechanisms. Several immunotherapy-based treatments have been developed to overcome this resistance and increase the long-term benefit. Most immunotherapies act on escape mechanisms like impaired antigen presentation, a decreased neoantigen repertoire and T-cell function, insensitivity to immune effector molecules, the tumour microenvironment and co-opting of alternative immune checkpoints [16]. In context of double ICI treatments, so far, the results in NSCLC are disappointing. The randomized phase III Checkmate 227 (NCT02477826) trial (nivolumab-ipilimumab) demonstrated prolonged 2-year OS compared to chemotherapy alone, independent of PD-L1 expression [17], albeit with a comparator arm (platinum doublet chemotherapy) which is now considered inferior [18]. On the other hand, the phase III MYSTIC (NCT02453282) and NEPTUNE (NCT02542293) trials (both durvalumab-tremelimumab) were reported negative for their primary endpoints [19, 20]. One option to improve OS is the addition of radiotherapy to ICI, as radiation might act synergistically with ICI on the immune system [21–23]. The added value of ICI has already been shown in stage III NSCLC, in which adjuvant durvalumab after concurrent chemoradiotherapy in patients with good PS resulted in an improved median PFS and OS, as well as an improved 3-year survival (66.3% versus 43.5%) [24, 25]. In stage IV NSCLC, early signals of efficacy have been observed. Albeit negative in the intention to treat population, the PEMBRO-RT phase II trial (NCT02492568) showed that combining pembrolizumab with stereotactic ablative radiotherapy (SABR) significantly increased the OS (12 months: 55% vs 36%, 18 months: 48% vs 28%) of PD-L1-negative NSCLC patients without increasing toxicity compared to pembrolizumab alone [26].

As the combination of radiotherapy and ICI still does not result in long-term benefit for most patients, the addition of new immunotherapy modalities to radiation should be explored. Based on personal communication, we know that L19-interleukin 2 (IL2) (darleukin; 15 MIO IU) has shown promising results in a phase I trial (NCT02086721) when combined with SABR in oligo-metastatic cancer patients. Two NSCLC patients (33%) are still without progression, respectively 3 and 4 years after treatment completion. Importantly, the combination did not result in grade 3 or higher toxicity. Based on these promising phase I data, we are performing a randomised phase II trial.

### Rationale

IL2 is a cytokine that plays an essential role in the activation of the innate and specific immune response [27, 28]. Unfortunately, the use of systemic administered IL2 is limited due to its serious acute toxicity profile which require intensive inpatient management [29]. Nevertheless, coupling IL2 to a tumour specific antibody, like L19, reduces the IL2 concentration in blood and increases the concentration in the tumour [30, 31], resulting in only low grade toxicity [32]. L19-IL2 is a fully human immunocytokine consisting of the human cytokine IL2 fused to the single-chain (scFv) human antibody fragment L19 targeting extra-domain B (ED-B) explicitly [33]. ED-B of fibronectin (FN) is a type III-FN domain, which can be inserted in the protein molecule by a mechanism of alternative splicing [34]. ED-Bcontaining fibronectin is a well-characterised marker of neo-angiogenesis and is expressed in the extracellular matrix surrounding newly formed blood vessels. As such, it is abundantly expressed around the vasculature of a variety of human cancers [35–37], while it is usually not present in healthy adult human tissues. It is known that in NSCLC, ED-B is expressed in the vast majority (~ 82%) of tumours [31, 38]. Darleukin represents a targeted form of IL2, capable of selective accumulation at the tumour site [39]. As such, this drug has the potential to boost selective anti-tumoural immune responses in patients with NSCLC. We have demonstrated in pre-clinical studies that L19-IL2, especially in combination with radiotherapy, results in improved disease control in lung and colorectal carcinoma models [21, 23]. Therapeutic responses were dependent on the irradiation dose, the target expression levels and are causally related to the presence of CD8^+^ cytotoxic T cells [21]. Best responses were found when administrating L19-IL2 after radiotherapy [40, 41]. Additionally, we have shown that this combination leads to improved local tumour control as well as tumour regression outside of the radiotherapy field and induces an immune-mediated memory effect preventing tumour-relapse after reinjection of tumour cells [23]. Furthermore, in pre-clinical Lewis Lung Carcinoma (LLC) models, we have observed that the synergy of radiotherapy with L19-IL2 is superior to the combination of radiotherapy with ICI. Interestingly, combining RT, L19-IL2 and ICI resulted in curative responses for this low immunogenic tumour model, which were associated with increased infiltration of NK and CD8^+^ T cells without any signs of toxicity [42]. Several trials with stage IV malignancies found 22.5 Mio IU as recommended L19-IL2 dose as monotherapy [32, 43, 44] or combined with dacarbazine [43, 44]. We recently received, based on personal communication, the safety results of L19-IL2 in the phase I trial (NCT02086721) given 15 Mio IU after SABR, as the combination of 22.5 Mio IU L19-IL2 with SABR resulted in more grade 3 toxicity. Therefore, the 15 Mio IU will be used in the current phase II trial protocol: ImmunoSABR (NCT03705403), designed to test the activity and efficacy of L19-IL2 (+ ICI treatment if SOC) following SABR in oligo-metastatic and conventional radiotherapy in poly-metastatic (maximum 10 metastatic sites) in NSCLC patients. A maximum of 10 lesions was defined, as patients without widespread metastases have low or acceptable toxicity when irradiating maximum 5 out of 10 lesions, and still have an active immune system to gain the best response on L19-IL2.

## Methods/design

**Fig. 1:**
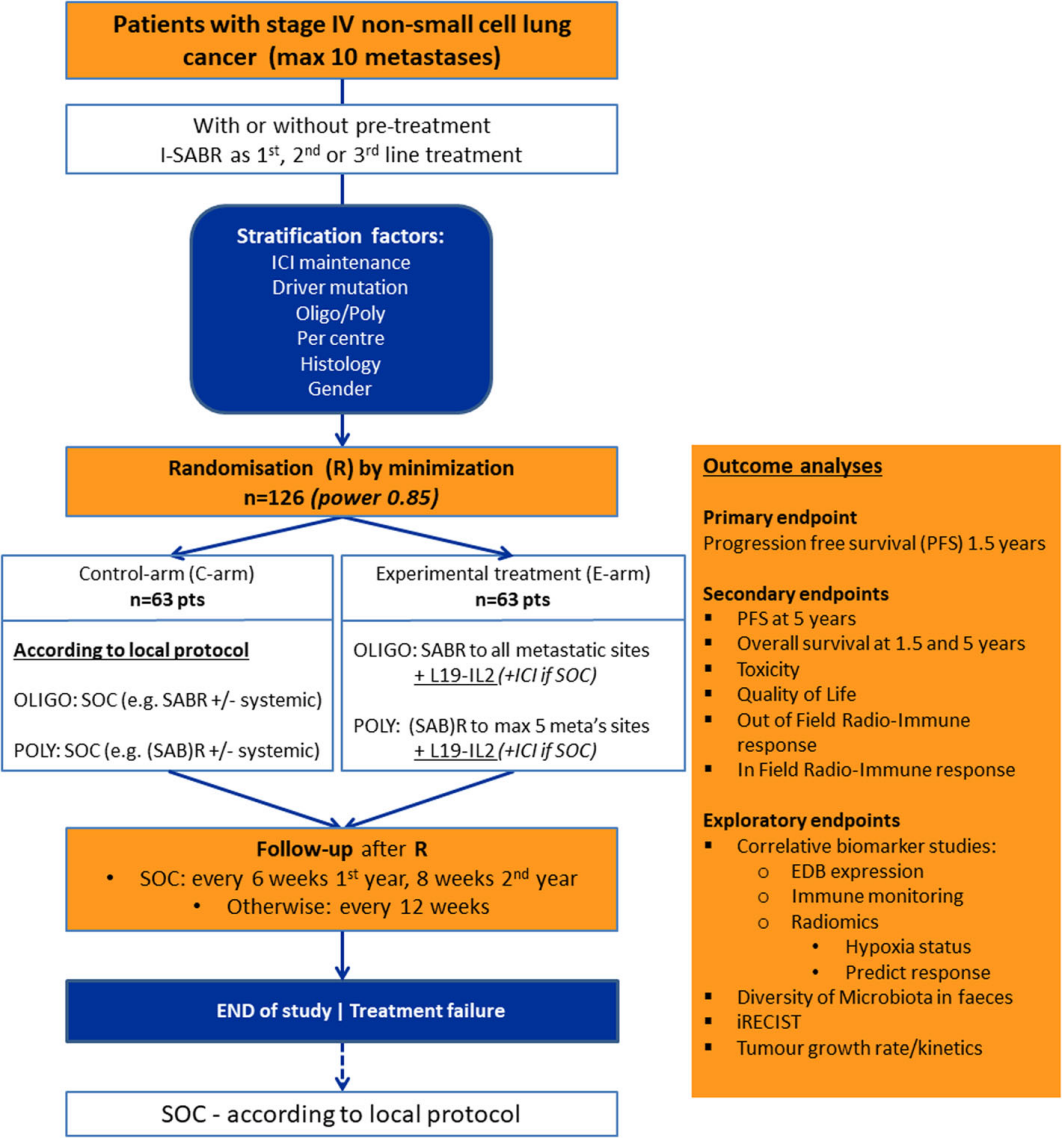
Study design of ImmunoSABR phase II trial

ImmunoSABR is a multicentre, randomised controlled open-label phase II clinical trial testing the hypothesis that the combination of (SAB)R and the immunocytokine L19-IL2 will increase the PFS in patients with limited metastatic NSCLC compared to SOC (including ICI in both arms if this is the SOC). After randomisation by minimisation, patients will be assigned either to the experimental arm (E-arm) or the control arm (C-arm) as described in Fig. 1.

### Study population

The trial will consist of one cohort of 126 adult patients with stage IV NSCLC. ImmunoSABR will act as 1st, 2nd or maximum 3rd line treatment, and will be different for patients with oligo-metastatic NSCLC (max 5 metastases [11]) and patients with poly-metastatic NSCLC (6–10 metastases). As SOC and OS differ between oligo-metastatic and poly-metastatic patients [7], oligo versus poly will be used as a stratification factor for randomisation. Also, ICI maintenance treatment, centre, histology (squamous versus non-squamous), gender and driver mutation (equally divide previously TKI treated patients with oncogenic drivers) will be added as stratification factors. Patients will be randomised into 2 arms, using randomisation by minimisation (ALEA software): E-arm or C-arm. The algorithm uses a random factor to prevent the possibility of upfront knowledge about the randomisation result. All data collected for this trial will be entered in and stored on an online clinical data management platform containing pre-structured electronic case report forms per patient visit.

### Sample size calculation

The expected 1.5-year PFS is minimally 15% in the C-arm and at least 35% in the E-arm. A sample size of 116 patients (58 patients per treatment arm) is needed to show this difference (20%) in PFS, using a Log-Rank test with a two-sided alpha of 0.05 and power of 85%. Patients will be evenly divided over the two arms during an accrual period of 2.5 years. Assuming a drop-out rate of 10%, a total of 126 patients (63 per arm) need to be included.

### Study treatment

**Fig. 2:**
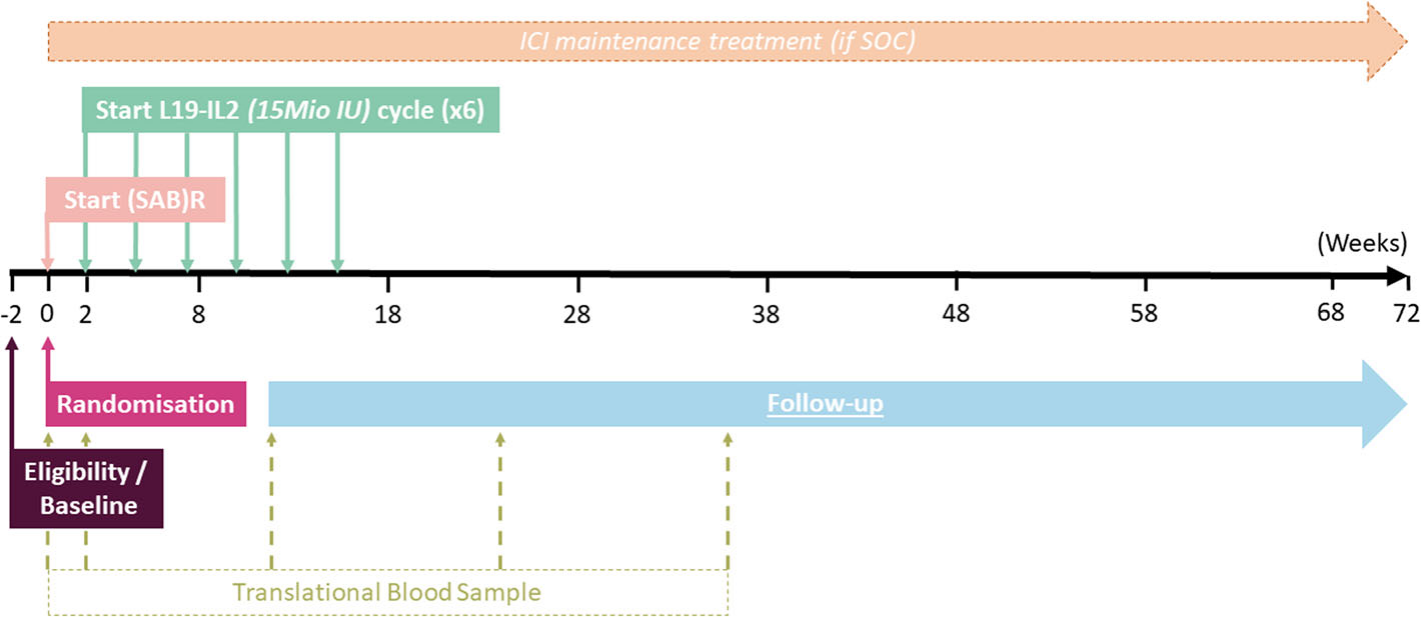
Timeline of study treatment and follow-up

A complete overview of the inclusion and exclusion criteria is described in supplementary Table 1. Main inclusion criteria are NSCLC, ≤10 metastatic lesions at baseline, world health organization preformance status (WHO PS) 0–1 and adequate bone marrow, liver and renal function. Most important exclusion criteria are a systemic infection, pregnancy, history of organ transplant, autoimmune disease and impaired/uncontrolled cardiovascular function. All potentially eligible patients have to sign the informed consent (ICF) (see supplementary Table 2 for the ICF) and will subsequently be examined with respect to medical history, physical examination, WHO PS, vital signs, height and body weight, general and viral blood tests (e.g. Creatinine, total protein, albumin, ALAT, ASAT, alkalic phosphatase, γ-GT, bilirubin, Hb, WBC and differentiation, platelets, PT and aPTT, HIV, HBV and HCV) and a pregnancy test in women of child-bearing potential. The participants fulfilling the afore-mentioned criteria will undergo baseline imaging (e.g. fluordeoxyglucose Positron Emission Tomography/Computed Tomography (FDG PET/CT) and brain Magnetic Resonance Imaging (MRI)) as performed in respective institutes before being randomised (t = 0) into the C-arm or E-arm, see Figs. 1 and 2.

The participants fulfilling the eligibility criteria will undergo a SOC baseline imaging, translational blood sampling, and other baseline requirements before being randomised (t=0) into the C-arm or E-arm. Shortly after randomisation, the first fraction of (SAB)R will be planned. L19-IL2 will start within 72 hours after last fraction. Every patient will receive 6 cycles, which consist each of 21 days. Simultaneously with these cycles, ICI will be administered if SOC. Follow-up for a period of 1.5 years after randomisation.

The SOC protocol will be applied for the patients within the C-arm, while having the same time points as the E-arm for translational samples and follow-up. Patients are allowed to have had front-line systemic therapy and/or treatment to the primary tumour prior to the trial, however, this is not obligatory. The participants in the E-arm will start with (SAB)R to maximum 5 metastatic lesions, according to a dose-fractionation regimen chosen by the research physician’s discretion. However, some study guidelines need to be taken into account: the minimum dose per fraction to the metastasis should be 7Gy for oligo-metastatic and 4Gy for poly-metastatic disease, with a relative biological effectiveness of in total 30-60Gy for oligo-metastatic and 8-35Gy for poly-metastatic disease, within every day or every second day between fractions [45–49]. The dose constraints for various critical organs suggested by the AAPM task group 101 should be respected [50]. To decrease the risk of radiation-induced lymphopenia, irradiation of large vessels (e.g. aorta), spleen and the heart should be avoided whenever possible [51]. Furthermore, irradiation scheduling should be organised to allow the first administration of L19-IL2 within 72 h after the last irradiation for patients randomised to the E-arm.

The participants in the E-arm will receive 6 cycles of L19-IL2 (and ICI maintenance treatment before every first L19-IL2 infusion of every cycle if SOC). Figure 2 and the supplementary Table 3 show a detailed timeline overview. One cycle L19-IL2 consists of three administrations of L19-IL2 at days 1, 3 and 5. Cycles are repeated every 3 weeks. At day 3 of each cycle, the quality of life (QoL) questionnaires EQ-5D, QLQ-C30 v3.0 and QLQ-LC13 will be filled out. Furthermore, before every L19-IL2 infusion, multiple tests will be performed, e.g. recording concurrent medication, blood tests, blood pressure, temperature, heart rate, WHO PS (0–1) and scoring adverse events with CTCAE v5.

During the first 3 h L19-IL2 infusion cycle, blood pressure, temperature and heart rate are assessed every 30 min, at the end of the administration and subsequently after 30 min, 1 h and 2 h following the administration. If no significant changes are seen in blood pressure, temperature and heart rate during the first 2 cycles, only end of infusion assessment will be required for subsequent cycles.

### Follow-up

In each centre, both arms will receive follow-up on the same time points, based on their SOC (every 6 weeks versus every 12 weeks). For both arms, there will be follow-up CT-scan(s) with IV contrast (slice thickness of ≤ 3 mm), WHO PS and Quality of Life questionnaires to be completed at least every 12 weeks. Also, translational blood samples are taken simultaneously with the planned CT-scans in week 12, 24 and 36. Blinded local radiological review will be performed for every follow-up scan to assess tumour response using RECIST (version 1.1, [52]) and exploratory iRECIST [53].

Response criteria are based on a set of measurable lesions identified at baseline as target lesions for RECIST evaluation, and followed until disease progression. If all lesions are irradiated, RECIST is less accurate. In case of doubt, we propose a panel discussion. Follow-up and treatment for patients progressing will be done according to the local standard protocols. Patients will be followed every 3 months to record OS, toxicity, QoL and adverse events for 1.5 years. The PFS and OS will be collected for a total of 5 years.

### Study parameters and endpoints

The main objective of the trial is to test if the combination of (SAB)R and the immunocytokine L19-IL2 (and ICI in case of SOC) will result in an increased PFS rate at 1.5 year after randomisation compared to the SOC, based on blinded radiological review (RECIST 1.1). The secondary objectives will be an assessment of 5-years PFS, 1.5-year and 5-year OS and 1.5-year CTCAE v5 toxicity grade, QoL, Out of Field Radio-Immune response (target(s) for RECIST analysis 1.1) and In-Field Radio-Immune response (non-target(s) for RECIST analysis). Exploratory analyses will be performed to investigate biomarkers (e.g. ED-B expression on tumour biopsies), diversity of the microbiota of faeces, CT radiomics and the changes of immunologic markers in repeated peripheral blood samples. The statistical methods are described in supplementary Table 4.

### Nature and extent of the burden and risks associated with participation, benefit and group relatedness

ICI, either alone for selected patients or in combination with chemotherapy, have become the SOC for most good PS patients with metastatic disease [5]. A specific population of metastasized NSCLC patients, namely those with oligo-metastatic disease, can obtain long-term survival with the addition of local ablative therapy [6, 7]. However, most patients (oligo- and poly-metastatic) do not obtain long-term survival and new treatment strategies are therefore needed. The ultimate aim of the combination of (SAB)R and L19-IL2 (with ICI if SOC) is to prolong PFS by inducing an immune response which would be able to control this systemic disease. Known/potential risks additional to the SOC treatment include:

#### L19-IL2 related side effects

Fever with chills, fatigue, nausea, vomiting, asthenia, (peripheral) oedema, skin rash, hyperhidrosis, chest pain, pruritus, elevated serum creatinine levels and pain at the tumour site. Signs of mild capillary leak syndrome and hypotension were found at the dose-limiting dosage level that is not administered in the current trial [32, 43, 54–58]. Most of the severe adverse events were seen in studies using a higher dose of L19-IL2 (22.5–30 Mio IU) compared to our phase I study dose of 15 Mio IU where no grade 3 or more toxicities occurred. We therefore expect, based on our experiences in the phase I trial, that the incidence and intensity of the adverse events are lower in the current phase II trial. Experiences from our phase 1 trial combined with published IL2-management guidelines [59–61] will be used as guidance when side effects occur.

#### (SAB)R related side effects

(SAB)R side effects are dependent on the location of the irradiated site. Toxicity is considered very low and the risk of radiation-induced lymphopenia will be decreased by avoiding, whenever conceivable, the irradiation of large vessels (e.g. aorta), spleen and the heart. Possible side effects are nausea, vomiting, diarrhoea for abdominal sites, local pain, discomfort and neuritis for soft tissue and bony sites and dyspnoea, cough, radiation pneumonitis and rib fractures for thoracic sites. Studies evaluating SABR to mixed oligo-metastatic sites report grade III toxicity rates below 12%, like bowel strictures, fatigue, dyspnoea, pain but also treatment-related death (e.g. radiation pneumonitis, pulmonary abscess) [62]. There might be an increased risk of immune-related toxicity for those patients receiving L19-IL2 and radiotherapy in combination with standard treatment pembrolizumab. Immune-related toxicity from standard of care pembrolizumab is rare, but can be serious and life-threatening [63]. Knowing that metastatic NSCLC is a mortal disease in the short-term, the potential burden seems proportional to the potential gain.

An independent Data Safety Monitoring Board (DSMB) has been established and will monitor patient recruitment, adverse effects reporting and data quality during the trial. A first safety analysis is planned after the first 15 patients treated with triple therapy. All Serious Adverse Events and Suspected Unexpected Serious Adverse Reactions (SUSAR) will be reported to the manufacture and following country specific and European Medicine Agency (EMA) guidelines.

### Translational research

The translational research will focus on immunological marker evaluation oriented towards both finding prognostic and predictive biomarkers that will indicate sensitivity to (SAB)R/L19-IL2 treatment. Also, blood (e.g. neutrophil counts [64], lymphocyte counts [65–67], LIPI score [68], circulating tumour DNA [69], cell-free tumour DNA [70] and immune cell subsets), tissue (e.g. ED-B expression, somatic mutations [71], non-synonymous mutation load [72, 73], PD(L)-1 expression, neutrophil and macrophage type 2 levels, Immune cell subsets) and faeces (diversity in microbiota) markers related to the importance of immunological checkpoints for the L19-IL2 interaction will be assessed. The samples will be collected (only in case of patient informed consent) and stored for 15 years, supplementary Table 5.

Radiomics is one of the most promising techniques that have the potential to improve cancer prognostication [65, 67]. In Radiomics, large numbers (1500+) of quantitative features such as tumour image intensity, (multi-scale) texture and the shape and size of a tumour are extracted from standard medical images (CT, PET, MRI) using (semi)automatic software. Radiomics enables identification of quantitative imaging biomarkers (QIBs) to quantify and classify tumour phenotypes and other disease parameters non-invasively. An exploratory correlative analysis will be conducted on the available PET-CT / diagnostic and simulation CT / MRI data obtained prior and during this trial. All the scans will be de-identified and collected every year and at the end of the trial [74]. We have three main hypotheses: (A) Studies suggest that the mutational landscape in tumours influences the response to immunotherapy, as higher non-synonymous mutation burden in tumours was associated with an improved objective response, durable clinical benefit and PFS [46, 47]. However, analysing these mutations in tissues is currently a complex and laborious process. We hypothesise that these phenotypic differences in a tumour can be characterised non-invasively by applying radiomics; the heterogeneous tumours will be more sensitive to immunotherapy than less heterogeneous tumours. (B) Hypoxia immunological niche: Hypoxia is a negative prognostic factor possibly causing resistance to immunotherapy [75]. We hypothesise that radiomics can be applied to assess tumour hypoxia and thus can be used as a predictive factor for treatment response. (C) We hypothesise that radiomics can more accurately assess the progression of the disease than RECIST.

The tumour growth rate (TGR) before and during treatment and variation per period will give insights in tumour development but also more information regarding treatment specific response. All available scans prior to (incl diagnostic scan) and during this trial will be used to visualise the TGR and determine hyperprogressive disease, based on [76].

## Discussion

A recent phase I study revealed the safety of the bimodal treatment but also indicated favorable treatment response in 2/6 patients being still progression-free for respectively 3 and 4 years. ImmunoSABR, a randomised phase II study, is the first randomised, investigator-initiated and intention to treat clinical trial that generates a reliable evidence base to change clinical practice from palliative to a curative treatment strategy in patients with limited metastatic NSCLC.

## Supplementary information

**Additional file 1: Supplementary Table 1.** In- and exclusion criteria.

**Additional file 2: Supplementary Table 2.** The informed consent form used for ImmunoSABR phase 2 trial.

**Additional file 3: Supplementary Table 3.** Schematic overview of the study and timeline.

**Additional file 4: Supplementary Table 4.** Statistical analysis study parameters and subgroup analyses.

**Additional file 5: Supplementary Table 5.** Overview of the translational research samples.

## Data Availability

Not applicable.

## Abbreviations

AAMP: American Association of Physicists in Medicine
AE: Adverse Event
aPD-(L)1: Anti-programmed Cell Death (Ligand) 1
ALAT: Alanine Amino Transferase
ASAT: Aspartate Amino Transferase
aPTT: activated partial thromboplastin time
C-arm: Control arm
CTCAE v5: Common Terminology Criteria for Adverse Events version 5
DSMB: Data Safety Monitoring Board
E-arm: Experimental arm
ED-B: Extra Domain B (of fibronectin)
EGFR: Epidermal Growth Factor
EORTC: QLQ-C30 European Organization for the Research and Treatment of Cancer Quality of Life Questionnaire C30
EQ5D: EuroQol- 5 Dimension
Fx: Fractions
γ-GT: gamma- glutamytransferase
Gy: Gray
Hb: hemoglobin
HBV: Hepatitis B virus
HCV: Hepatitis C virus
HIV: Human immunodeficiency virus
IFRI: In Field Radio-Immune
iRECIST: immuno- Response Evaluation Criteria in Solid Tumors
LC13: 13 Lung cancer specific questions
LVEF: Left ventricular ejection fraction
MIO: IU million international unit
NSCLC: Non-Small Cell Lung Cancer
OFRI: Out of Field Radio-Immune
OS: Overall Survival
PCM: Paracetamol
PI: Principal investigator
PFS: Progression Free Survival
PT: Prothrombin time
PTV: Planning Target Volume
QoL: Quality of Life
RECIST: Response Evaluation Criteria in Solid Tumors
RT: Radiotherapy
SABR: Stereotactic Ablative Body Radiotherapy
SAE: Serious Adverse Event
SOC: Standard of Care
SOP: Standard Operating Procedure
SUSAR: Suspected Unexpected Serious Adverse Reaction
TGR: Tumour growth rate
WBC: White Blood Count
WHO PS: World health organization preformance status
