# Supplementary material for: Stereotactic ablative body radiotherapy (SABR) combined with immunotherapy (L19-IL2) versus standard of care in stage IV NSCLC patients, ImmunoSABR: a multicentre, randomised controlled open-label phase II trial
Source: BMC Cancer. 2020 Jun 15;20:557. doi: 10.1186/s12885-020-07055-1 (PMC7296663; doi:10.1186/s12885-020-07055-1)
Supplement: Supplementary file 1 — Additional file 1: Supplementary Table 1. In- and exclusion criteria. [file 12885_2020_7055_MOESM1_ESM.pdf]

Supplementary Table 1. In- and exclusion criteria

Inclusion	Exclusion
Specific for:<br><u>Oligo-metastatic disease</u><br>• Maximum 5 metastases.<br><br><u>Poly-metastatic disease</u><br>• Minimal 6 - maximum 10 metastases.<br>• At least one measurable lesion (according to RECIST 1.1) that has no overlap with the PTV of the lesion subjected to radiotherapy.<br><br>*<u>Both groups</u>*:<br>• Stage IV NSCLC, regardless of the PDL1 status. (SOC baseline imaging within 6 weeks prior to randomisation.)<br>• Prior cancer treatments are allowed but must be discontinued for at least 4 weeks before randomisation (except aPD(L)1 treatment).<br>• Maximum two brain lesion with a total cumulative diameter of 5cm is allowed.<br>• Age of 18 years or older.<br>• WHO Performance Status 0-1.<br>• The patient is capable of complying with study procedures.<br>• The life expectancy of at least 12 weeks.<br>• Adequate bone marrow function: Absolute Neutrophil Count (ANC) of $\geq 1.0 \times 10^9$ /L, platelet count $\geq 100 \times 10^9$/L, Haemoglobin (Hb) $\geq 6 \times 10^9$ /L (it is allowed to give a blood transfusion if Hb is initially too low).<br>• Adequate hepatic function: total bilirubin ≤ 1.5 x upper limit of normal (ULN) for the institution; ALT, AST, and alkaline phosphatase ≤ 2.5 x ULN for the institution or ≤ 5 in case of liver metastasis.	• More than 10 metastatic lesions.<br>• More than 2 brain metastatic lesions.<br>• Two brain metastases with a cumulative diameter larger than 5 cm.<br>• Whole-brain radiotherapy (WBRT) is not allowed during the study.<br>• Patients with non-infectious pneumonitis, uncontrolled thyroid disease, pleuritis, pericarditis and peritonitis carcinomatosis.<br>• Patients who are already actively participating in another study.<br>• Simultaneous radiation on the primary tumour and metastatic lesion(s) during the trial.<br>• Previous radiotherapy to an area that would be re-treated by (SAB)R, resulting in overlap of the high dose areas.<br>• Maintenance therapy with aPD(L)1 treatment combined with chemotherapy is not allowed during treatment ((SAB)R and L19-IL2 cycles).<br>• Other active malignancy or malignancy within the last 2 years (except localised skin basal/squamous cell carcinoma, non-muscle invasive carcinoma of the bladder or in situ carcinoma from any site).<br>• History of allergy to intravenously administered proteins / peptides / antibodies / radiographic contrast media.<br>• HIV positive; active HIV infection, or active hepatitis B or C.<br>• Systemic treatment with either corticosteroid (>10 mg daily prednisone equivalents) or Interferon alpha or

• Adequate renal function: creatinine clearance of at least 40 ml/min.<br>• Negative serum pregnancy test for females of childbearing potential.<br>• Contraception requirements *[72]*: during the screening to six months following the last study drug administration and 4 months after the last dose of ant-PD(L)1 maintenance treatment.<br>• Signed and dated written informed consent.	immunosuppressive medications within 14 days prior to randomisation or during the trial. Topical or inhalation steroids are allowed. If a patient needs to take unexpectedly immunosuppressive medication during the trial, it will be allowed but decreasing the dose as soon as possible is strongly advised.<br>• Prior history of organ transplant, including allogeneic stem cell transplant.<br>• History or evidence of active autoimmune disease.<br>• Acute or sub-acute coronary syndromes within the last year, acute inflammatory heart disease, heart insufficiency NYHA > 2, or irreversible cardiac arrhythmias.<br>• A known impaired cardiac function defined as left ventricular ejection fraction (LVEF) < 50 % (or below the study site's lower limit of normal) as measured by MUGA or ECHO.<br>• Uncontrolled hypertensive disease; (systolic blood pressure (SBP) ≥160 mmHg or diastolic blood pressure (DBP) ≥100 mmHg during two measurements).<br>• Severe diabetic retinopathy (neoangiogenesis targeted by L19 outside the tumour).<br>• Major trauma, including oncologic surgery.<br>• Any underlying mental, medical or psychiatric condition which in the opinion of the investigator will make administration of study drug hazardous or hinder the interpretation of study results. Unstable or serious concurrent uncontrolled medical conditions.<br>• Pregnancy or breast feeding.
