# Supplementary material for: Stereotactic ablative body radiotherapy (SABR) combined with immunotherapy (L19-IL2) versus standard of care in stage IV NSCLC patients, ImmunoSABR: a multicentre, randomised controlled open-label phase II trial
Source: BMC Cancer. 2020 Jun 15;20:557. doi: 10.1186/s12885-020-07055-1 (PMC7296663; doi:10.1186/s12885-020-07055-1)
Supplement: Supplementary file 2 — Additional file 2: Supplementary Table 2. The informed consent form used for ImmunoSABR phase 2 trial. [file 12885_2020_7055_MOESM2_ESM.pdf]

*Study Code:* *Site ID Code:* *Participant identification number:*

| | | | | | | | | |
|---|---|---|---|---|---|---|---|---|
| | | | | | | | | |

**CONSENT FORM**

**Phase II Study of the Combination of L19-IL2 immunotherapy and (stereotactic ablative) radiotherapy in Patients Metastatic Non-small Cell Lung Cancer**

*Name of Research Physician:*

*Tel N° of Research Physician*

***If you agree, please initial box***

| | |
|---|---|
| 1. I was asked to participate in the clinical study ImmunoSABR. Dr. ________________________ explained the purpose of the study, what it involves and the practical arrangements. | |
| 2. I confirm that I have read the information sheet dated .............................. (version……) for this study. I was informed about participating and understand what it involves. I was informed about the possible benefits and disadvantages (side effects, risks, discomforts) which may occur during the study. I have had the time and the opportunity to ask questions and to reflect about the answers provided. | |
| 3. I understand that my participation is voluntary and that I am free to withdraw at any time without giving any reason, without my medical care or legal rights being affected. | |
| 4. I understand that relevant sections of my medical notes and data collected during the study may be looked at by individuals from the Sponsor, from the coordination center "[name CRO]", from regulatory authorities, where it is relevant to my taking part in this research. I give permission for these individuals to have access to my records. Personal information will be treated strictly confidentially by them. | |
| 5. I understand that during my participation in the study data will be collected about me and that the research physician and the sponsor guarantee the confidentiality of this information. | |
| 6. I agree that my personal data are processed according to the stipulations described in the section about guaranteeing the confidentiality (section X in Patient Information). I also agree with the transfer and processing of my coded data in countries other than [COUNTRY]. | |
| 7. I agree to my General Practitioner being informed of my participation in the study. | |
| 8. I agree that the sponsor will contact regularly my research physician up to 5 years after randomisation to ask about my medical condition. | |
| 9. I hereby agree that blood/tissue/stool samples are drowned/stored for further research. Tick if approved:<br>Blood samples for future research (mandatory):<br>☐ I **give permission** to the collection of blood samples for future research.<br>☐ I **give permission** to the storage (in pseudonymised form) of blood samples in [Institute name].<br>☐ I understand that if I do not agree with the collection and storage of blood samples for future research, I **cannot** participate to the study. | |

*Study Code:* *Site ID Code:* *Participant identification number:*

| | | | | | | | | |
|---|---|---|---|---|---|---|---|---|
| | | | | | | | | |

| | |
|---|---|
| Stool samples for future research (mandatory) :<br>☐ I **do** agree that a stool sample is collected once in the course of this study.<br>☐ I **do not** agree that a stool sample is collected once in the course of this study.<br>☐ I understand that if I do not agree with the collection of stool samples for future research, I still **can** participate to the study.<br><br>Biopsy samples for future research (optional):<br>☐ I **do** agree that new tumour tissue is taken from the tumour or metastasis for future research and I give permission to the storage (in pseudonymised form) of the collected **new tumour tissue** in [Institute name].<br>☐ I **do not** agree that new tumour tissue is taken from the tumour or metastasis for future research.<br><br>☐ I **do** agree that previously taken tumour tissue is retrieved from the archives/pathology department for future research and I give permission to the storage (in pseudonymised form) of the collected **tumour tissue of archived material** in the [Institute name].<br>☐ I **do not** agree that previously taken tumour tissue is retrieved from the archives/pathology department for future research.<br><br>☐ I understand that if I do not agree with the collection and storage of biopsy samples for future research, I still **can** participate to the study.<br><br>Scans(MRI/PET-CT/CT) from diagnose to start study (optional):<br>☐ I **do** agree that previously taken scans in relation to the disease are collected from the archives/radiologist department, for future research, and I give permission to the storage (in pseudonymised form) of these scans at [Institute name].<br>☐ I **do not** agree that previously taken scans in relation to the disease are collected from the archives/radiologist department, for future research.<br><br>☐ I understand that if I do not agree with the collection and storage of the scans for future research, I still **can** participate to the study.<br><br>Scans(MRI/PET-CT/CT) collected during the study (mandatory):<br>☐ I **do** agree that scans during the study are collected, for future research, and I give permission to the storage (in pseudonymised form) of these scans at [Institute name].<br><br>☐ I understand that if I **do not** agree with the collection and storage of the scans for future research, I **cannot** participate to the study. | |
| 10. I understand that I **cannot** be pregnant/ becoming pregnant / breastfeeding during the treatment and if I am a man, that my wife/partner cannot be pregnant/becoming pregnant / breastfeeding during my treatment.<br>If I am a woman maximum 2 pregnancy tests will be performed if necessary, at the start and at the end of the treatment. | |
| 11. I received a copy of the information for the participant and the informed consent. | |

*Study Code:* *Site ID Code:* *Participant identification number:*

| | |
|---|---|
| 12. I hereby declare that I voluntarily participate in the above scientific research, the data of which will be stored for X years. | |
| 13. I agree to take part in this study. | |

____________________ ____________________ ____________________

*Name of Participant* *Signature* *Date*

**Research physician**
I (the undersigned) confirm that I have explained the nature, the objective and the risks of the aforementioned scientific study to the said patient and that I have informed the patient to the best of my ability about the study and that I can assume that the patient understood the information.
Name of the research physician: Signature: Date:

.................................................... ............................................ ...............................

**Legal representative** (if applicable):
I declare that I was informed about the question to take a decision about participating in a clinical study by the person I represent in his best interest, taking into account his or her possible wish. My consent applies to all items included in the consent form for the participant.
Name of the legal representative: Signature: Date:

.................................................... ............................................ ...............................

**Witness / Interpreter*** (If applicable)
I was present during the entire information provision process to the patient and I confirm that the information about the objectives and procedures of the study was properly provided, that the participant (or his legal representative) are most likely to have understood the study and that the consent for participation was given voluntarily.
Name of witness/interpreter*: Signature: Date:

.................................................... ............................................ ...............................
** A witness is required when the patient is not capable to read (blind, illiterate). An impartial witness must be present for the entire duration of the interview about the informed consent.*
