# Supplementary material for: Stereotactic ablative body radiotherapy (SABR) combined with immunotherapy (L19-IL2) versus standard of care in stage IV NSCLC patients, ImmunoSABR: a multicentre, randomised controlled open-label phase II trial
Source: BMC Cancer. 2020 Jun 15;20:557. doi: 10.1186/s12885-020-07055-1 (PMC7296663; doi:10.1186/s12885-020-07055-1)
Supplement: Supplementary file 3 — Additional file 3: Supplementary Table 3. Schematic overview of the study and timeline. [file 12885_2020_7055_MOESM3_ESM.pdf]

Screening phase<br><br>C and E-arm	**PART I: Standard of care:**<br>*Oligo - SABR*<br>*Poly - (SAB)R*<br><br>Day X + 1-14days (depends on total fx)	Last day Friday (72h max before Immuno.)	**PART II: Immunotherapy (x6 cycles)**<br><br>Day Y +3 (Monday) // Y+24 days // +45 // +66 // +87 // +108	Day Y +5 (Wednesday) // Y+26 days // +47 // +68 // +89 // +110	Day Y + 7 (Friday) // Y+28 days // +49 // +70 // +91 // +112	**Follow-up*** *(most important timepoints showed)*<br><br>Start counting at randomisation day (R)
Before trail inclusion (min1-5days)	Start study	Day *Y	Day Y + 3	Day Y + 5	Day Y + 7	Day R+12wks
Eligibility: known inclusion/exclusion criteria, (SAB)R, aPD(L)1 maintenance?		Last (SAB)R fx<br>Follow-up visit	***Pre-injection***<br>Blood tests	***Pre-injection***<br>*#Blood tests*	***Pre-injection***<br>*#Blood tests*	**QOL** +WHO + adverse events<br>CT-scan
Day 1	Day X	! Blood for translational research (in case of allocation to arm E this bloodsample can be taken together with the bloodtest on first injection day)	WHO<br>Concurrent therapy & medication, PCM 1gr 1h prior, weight	**QOL** + WHO<br>Concurrent therapy & medication, PCM 1gr 1h prior	WHO<br>Concurrent therapy & medication, PCM 1gr 1h prior	**!** Blood for translational research
Informed consent<br>Demographics, medical history, physical examination, vital signs (BP, P, T) height and body weight. Assessment of concurrent therapy. Blood sampling (incl pregnancy test) Histology, collection of stratification factors, SOC baseline imaging (<6w).<br><br>Evaluation of adverse events	Start (SAB)R fx(n)					
	Day X + <14		**During-3h-injection:**<br>Infusion cycle 30min (BP, T, HR, BR), Adverse events	**During-3h-injection:**<br>Infusion cycle 30min (BP, T, HR, BR), Adverse events	**During-3h-injection:**<br>Infusion cycle 30min (BP, T, HR, BR), Adverse events	Day R + ~18wks *(Tumour response evaluation (>4weeks after last day cycle 6))*
Investigator decision ECG+LVEF	Follow-up visit (can be the same as on day Y)		**Post-injection:**<br>Infusion cycle (BP, T, HR, BR) at 30min, 1h and 2h, Adverse events	**Post-injection:**<br>Infusion cycle (BP, T, HR, BR) at 30min, 1h and 2h, Adverse events	**Post-injection:**<br>Infusion cycle (BP, T, HR, BR) at 30min, 1h and 2h, Adverse events	**QOL** +WHO + adverse events<br>CT-scan:CR/PR/SD/PD
Day 3- ~8<br>*(signed informed consent and Eligible conform in&exclusion)*				ONLY FOR ARM "E"		Day R + 24wks
**Randomisation (R)**						CT-scan<br>**QOL** +WHO + adverse events<br>**!** Blood for translational research
E-arm>> Plan Planning CT >> Start developing irradiation plan						Day R + 36wks
**Translational blood**<br>***Stool sample + optional biopsy + archived biopsy***						CT-scan<br>**QOL** +WHO + adverse<br>**!** Blood for translational research
Baseline **QOL**, Baseline WHO						Day R + 48wks //+54wks //+62wks //+70wks //+78wks
**Irradiation plan finished**						**QOL** +WHO + adverse events<br>CT-scan

Legend - C an E-arm

X: Day were first fx of SABR will be given
Y: Last day of the last fx of SABR
R: Day were randomisation took place

CR: Complete response; disappearance all target and non-target lesions & normalization of tumour markers
PR: Partial response; at least a 30% decrease in target lesions, Non target lesions non-PD
SD: Stable disease
PD: Progressive disease; at least a 20% increase and/or new lesions

QOL: Quality of life questionnaires (C30, LC13 and EQ5D)

#Blood tests: blood test is only planned if it is the investigator's decision.

*** Be aware that only most important time points are presented. Other time points e.g. 6wks interval (if SOC) are not shown.**

Be aware: E-arm patients will receive FU-scans (incl contrast) during L19-IL2 cyclus. FU-scans 1.5 weeks after L19-IL2 infusion but befor start new cycle!

BP: blood pressure, ECG: electrocardiogram, fx: fractions, gr: gram, h: hour, HR: heart rate, LVEF: left ventricular ejection fraction, P: puls, R: randomisation, T: temperature, WHO: world health organization Performance Status, wks: weeks.
