# Supplementary material for: Stereotactic ablative body radiotherapy (SABR) combined with immunotherapy (L19-IL2) versus standard of care in stage IV NSCLC patients, ImmunoSABR: a multicentre, randomised controlled open-label phase II trial
Source: BMC Cancer. 2020 Jun 15;20:557. doi: 10.1186/s12885-020-07055-1 (PMC7296663; doi:10.1186/s12885-020-07055-1)
Supplement: Supplementary file 4 — Additional file 4: Supplementary Table 4. Statistical analysis study parameters and subgroup analyses. [file 12885_2020_7055_MOESM4_ESM.pdf]

STATISTICAL ANALYSIS

ImmunoSABR is a controlled randomised open-label phase II trial which aims to demonstrate an absolute increase in progression-free survival at 1.5 years after randomisation (primary endpoint). The trial consists of two arms: the C-arm and E-arm. In the E-arm patients will receive radiotherapy using (SAB)R to maximal 5 metastatic lesions and 3 days later start with the first of 6 cycles of the immunocytokine L19-IL2. All patients with Poly-metastatic disease (up to 10 metastases), received either no previous treatment or first or second line chemo and/or immunotherapy treatment before enrolment in the study.

The randomisation by minimization will take into account the following stratification factors:

Centre, Oligometastatic vs Poly-metastases, maintenance with Anti-PD(L)1 treatment (yes/no), gender (male vs female), histology (squamous vs other), driver mutation (EGFR, ALK, ROS, MET or Other/unknown).

**Primary study parameter**	The primary endpoint is a difference in PFS between the E-arm (with L19-IL2) and the C-arm (without L19-IL2). PFS will be determined as the time between randomisation and disease progression, according to RECIST 1.1, or death due to any cause. Subjects will be followed until either (a) the terminal event occurs, or (b) they drop out of the study. If the event does not occur during the period that the patient is followed, the patient will be censored. Comparison between control and experimental arm will be made using the Log-Rank statistic.<br>In addition, a multivariate Cox proportional hazards (CPH) model will be used to assess the effect of treatment arm on the primary outcome, corrected for gender, total tumour burden (Oligo vs. Poly), and histology. Hazard ratios (HR) and their respective 95% confidence intervals will be reported. The effect of study centre will be taken into account by adding centre as a random effect.

**Secondary study parameter(s)**	• Overall survival will be estimated by Kaplan-Meier analysis for each treatment arm and will be compared between arms using a Log-Rank test. Patients that are still alive at the last follow-up will be censored.<br>• Toxicity will be analysed for each treatment arm. Descriptive statistics for toxicity will be provided in tables. Logistic regression analyses to compare toxicity between the treatment arms will be performed by categorizing toxicity grade into severe (≥3) and not severe (≤2).<br>• Quality of life measurements will be taken at baseline and at various time points during the treatment. Changes over time in QoL measurements will be estimated for each treatment arm using mixed measures repeated modelling. Treatment, gender, metastatic disease (Oligo versus Poly-metastases), and an interaction term for treatment x visit will be entered into the model as fixed effects. Subject will be included as a random effect with an unstructured (UN) covariance matrix for the repeated measures within subject. Centre or hospital will also be entered as a random effect.<br>• The OFRI (abscopal) response will be reported as the number and percentage of patients that developed a measurable response according to RECIST 1.1 outside of the radiotherapy field (outside the PTV). This will be reported for all assessments, i.e. every 6 weeks for the first year and 8 weeks for the second year if SOC, otherwise at least every 12 weeks, according to the study protocol.<br>• The IFRI (in field radio-immune) response will be reported as the number and percentage of patients that developed a measurable response according to RECIST 1.1 inside of the radiotherapy field (inside the PTV). This will be reported for all assessments, i.e. every 6 weeks for the first year and 8 weeks for the second year if SOC, otherwise at least every 12 weeks, according to the study protocol.
**Subgroup analyses**	Subgroup analyses will be performed for 1) specific driver mutations, and 2) Oligometastatic disease and Poly-metastatic disease to assess

	the effect on PFS and overall survival. The Kaplan Meier method and Cox regression will be used for these subgroup analyses.
**Other study parameters**	Before and during treatment, blood and possibly tumour tissue are collected for correlation of EDB expression, immune infiltrate profiling, and lymphopenia, with outcome measures such as PFS, OS, OFRI, and IFRI. Descriptive statistics will be reported and, if appropriate, differences will be tested for statistical significance using T-tests for continuous outcomes, Chi-square tests for categorical data, and Kaplan Meier Log-Rank tests for time-to-event outcomes. Because tissue will not be available for all patients, the power to detect statistically significant differences is expected to be low. In addition, associations between the listed study parameters and the outcome measures are currently unknown. Therefore, all comparisons will be purely exploratory.
**Missing Parameters**	For the primary outcome as well as the secondary outcomes an intention-to-treat and a per-protocol analysis will be performed. If applicable, a worst-case scenario will be used to impute missing outcome values. However, the time-to-event analysis implicitly handles missing information by censoring these patients.
