# Supplementary material for: Stereotactic ablative body radiotherapy (SABR) combined with immunotherapy (L19-IL2) versus standard of care in stage IV NSCLC patients, ImmunoSABR: a multicentre, randomised controlled open-label phase II trial
Source: BMC Cancer. 2020 Jun 15;20:557. doi: 10.1186/s12885-020-07055-1 (PMC7296663; doi:10.1186/s12885-020-07055-1)
Supplement: Supplementary file 5 — Additional file 5: Supplementary Table 5. Overview of the translational research samples. [file 12885_2020_7055_MOESM5_ESM.pdf]

Sample	Quantity	Time point	Reason(s)
**Blood samples**	EDTA (10ml)	Baseline, during treatment	Collection of EDTA plasma and cfDNA
	Heparin plasma (50ml)	Baseline, after last (SAB)R fx, 12, 24, and 36 weeks	Extraction of isolated PMBC's and plasma
**Paraffin-embedded tissue**	20 Slices (two per slide)<br>2 Slices for RNA-later	Baseline	Immunological staining<br>ED-B staining
**Stool sample**	1 Kit	Baseline	Microbiota
**(Optional) biopsy**	1-2 Biopsies in RNA-later	Baseline	Immunological marker

EDTA: Ethylenediaminetetraacetic acid, cfDNA: cell-free DNA, PBMC: peripheral blood mononuclear cell, ED-B: extra-domain B.
